# Third chromosome candidate genes for conspecific sperm precedence between *D. simulans *and *D. mauritiana*

**DOI:** 10.1186/1471-2156-11-21

**Published:** 2010-04-13

**Authors:** Lisa Levesque, Barb Brouwers, Vignesh Sundararajan, Alberto Civetta

**Affiliations:** 1 Department of Biology, University of Winnipeg, Winnipeg, Manitoba, R3B 2E9, Canada; 2 Department of Biochemistry & Medical Genetics, University of Manitoba, Winnipeg, Manitoba, R3E 0J9, Canada

## Abstract

**Background:**

Male - female incompatibilities can be critical in keeping species as separate and discrete units. Premating incompatibilities and postzygotic hybrid sterility/inviability have been widely studied as isolating barriers between species. In recent years, a number of studies have brought attention to postmating prezygotic barriers arising from male - male competition and male - female interactions. Yet little is known about the genetic basis of postmating prezygotic isolation barriers between species.

**Results:**

Using *D. simulans *lines with mapped introgressions of *D. mauritiana *into their third chromosome, we find at least two *D. mauritiana *introgressions causing male breakdown in competitive paternity success. Eighty one genes within the mapped introgressed regions were identified as broad-sense candidates on the basis of male reproductive tract expression and male-related function. The list of candidates was narrowed down to five genes based on differences in male reproductive tract expression between *D. simulans *and *D. mauritiana*. Another ten genes were confirmed as candidates using evidence of adaptive gene coding sequence diversification in the *D. simulans *and/or *D. mauritiana *lineage. Our results show a complex genetic basis for conspecific sperm precedence, with evidence of gene interactions between at least two third chromosome loci. Pleiotropy is also evident from correlation between conspecific sperm precedence and female induced fecundity and the identification of candidate genes that might exert an effect through genetic conflict and immunity.

**Conclusions:**

We identified at least two loci responsible for conspecific sperm precedence. A third of candidate genes within these two loci are located in the 89B cytogenetic position, highlighting a possible major role for this chromosome position during the evolution of species specific adaptations to postmating prezygotic reproductive challenges.

## Background

The Biological Species Concept defines species as actual or potential interbreeding individuals that are reproductively isolated from others [1]. Any type of male - female reproductive incompatibilities can therefore be critical in keeping species as separate and discrete units. Premating behavioral incompatibilities can arise as a consequence of different competitive strategies between males, females avoiding male mating signals, or male rejection of heterospecific females [2-4]. Postmating postzygotic mechanisms of isolation, such as hybrid inviability or sterility, have also been widely studied as reproductive isolation barriers between species [5].

In species where females mate with multiple males, a different arena is set for male - male competition and male - female interactions. There is evidence that polyandry imposes numerous pressures on the coevolution of males and females, not only in terms of premating signal-response exchanges, but also primary genital morphology [6-9], sperm and female sperm storage organ morphology [10-12] and even postmating chemical cues [13-15]. Coevolution under the competitive pressures imposed by multiply mating females can contribute to reproductive isolation by promoting genetic divergence between populations, possibly involving reinforcement [16-18].

One form of postmating prezygotic isolation is conspecific sperm precedence (CSP); defined as the preferential utilization of conspecific sperm for fertilization, when females have been inseminated by both conspecific and heterospecific males. Conspecific sperm or gamete precedence has been studied in a wide variety of invertebrates, vertebrates, and plants [19]. One of the best examples is from studies of natural populations of the cricket genus *Allonemobius*, where two closely related species capable of mating among heterospecifics in nature are isolated by noncompetitive gametic isolation as well as preferential fertilization by conspecifics [20,21].

In *Drosophila melanogaster*, females willingly remate in both laboratory and wild populations [22,23], generating an opportunity for sperm competition. The outcome of sperm competition in Drosophila is commonly measured as second male paternity success in double mating experiments and is influenced by complex male × male interactions [24], male × female interactions [25], male × male × female interactions and trade offs with other postmating reproductive traits [26,27]. These interactions allude to the complexity of the mechanisms underlying sperm competition outcomes and also to the intricate nature of male - female coevolution in Drosophila.

The within and between sexes interactions and trade offs are likely to contribute to the large amount of intraspecific variation in first (*P1*) and second (*P2*) male paternity success, though the second male typically sires the majority of progeny (more than 60%) [28,29]. This pattern is also seen in heterotypic crosses involving *D. melanogaster *cosmopolitan and Zimbabwe races but disappears when a female mates with a conspecific and a heterospecific male. Regardless of the order of mating, the conspecific male sires the majority of progeny [30,31]. Studies using females singly mated to heterospecific males or doubly mated to a sterile conspecific and a fertile heterospecific male have identified both sperm incapacitation and sperm displacement as barriers to heterospecific fertilization success [32,33]. However, a detailed understanding of CSP in Drosophila is lacking owing largely to experimental difficulties to discriminate heterospecific and conspecific sperm within the reproductive tract of a doubly mated female.

Using a variety of approaches, the genetic basis of intrinsic postmating isolation barriers between species has been thoroughly studied. Studies of postzygotic isolation have led to the identification of hybrid sterility and hybrid inviability genes [5]. However, only two studies have previously attempted to map genes responsible for CSP. Using *D. simulans *- *D. sechellia *introgression lines, significant QTLs were detected on the second and third chromosome only when using low stringency statistical thresholds [34]. Another QTL approach using reciprocal F2 backcross females of crosses between two crickets, *Allonemobius fasciatus *and *A. socius*, mated to males of the two species found several unlinked markers associated with either enhancing or reducing conspecific male paternity success [35].

Here we use sixty *D. simulans *lines each with a single *D. mauritiana *third chromosome mapped introgression (IG lines) [36] in a double mating experimental design. Heterozygous males from each of the IG lines were competed as second male against males of a pure *D. simulans *strain. For some IG lines, the ability of males to sire progeny when second to mate did not differ significantly from that of *D. mauritiana *males and so were identified as poor sperm competitors. Males from these IG lines share a *D. mauritiana *introgression and it appears that at least two regions, one in the 77B to 84B and the other in the 88B to 92E cytogenetic map range, are sufficient to cause CSP. We identify eighty one broad-sense candidate genes within these chromosomal regions on the basis of male reproductive expression and Gene Ontology searches using broad search terms associated with sex and reproduction. The list was narrowed down by looking for regulatory and structural changes. On the basis of differential gene expression in the male reproductive tract of *D. simulans *and *D. mauritiana*, we narrowed down the list of candidates to five genes, three of them located within the 89B chromosomal position. Using an evolutionary approach that fits models specifying different rates of nonsynonymous and synonymous substitutions within coding sequences and along the *D. simulans *and *D. mauritiana *lineages, we identified an additional ten candidate genes, two in the 89B map position, that have undergone species specific adaptations. While the role of all fifteen candidate genes is unclear, gene-gene interactions and the coregulation of a gene cluster within the 89B cytogenetic map position appear to have been critical during the evolution of species specific adaptations to competitive male paternity success. *Mst89B *is a particularly interesting gene within the 89B position, because it has been shown to indirectly interact with *Acp62F *[37], a gene known to influence male sperm competitive ability in *D. melanogaster *[29,38].

## Results

### At least two loci cause breakdown in second male paternity success

**Figure 1:**
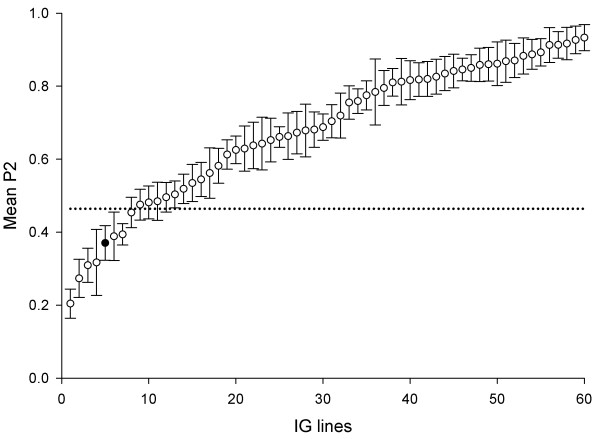
**Average second male paternity success (*P2*) for males from 60 different *D. simulans *introgressed (IG) lines**. For each line we show averages and standard errors. The average P2 score of *D. mauritiana *is shown as a black circle. The upper bound of the 95% confidence interval of *D. mauritiana *average *P2 *is shown as a dotted line.

**Figure 2:**
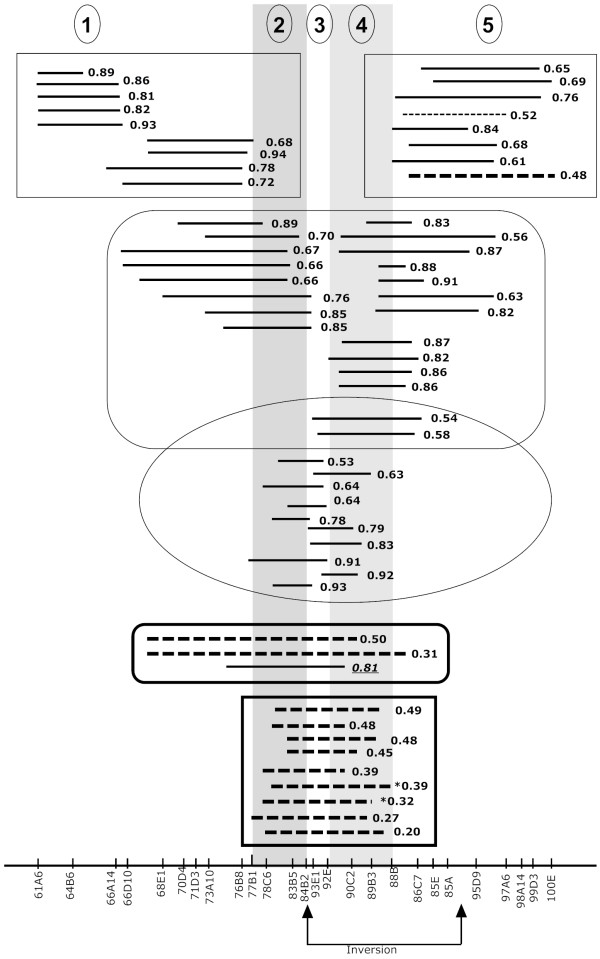
**Map position for the 60 *D. mauritiana *introgressions within the *D. simulans *third chromosome tested in this study**. Highlighted in grey are the minimum two loci introgressions causing a breakdown in second male paternity success and chromosome sections are divided by the two loci into five regions. Different shapes are used to box introgressions as spanning only regions 1 or 5 (rectangles), 2 and 3 or 3 and 4 (round edge rectangle), 2 and 3 or 3 and 4 (circle), at least four regions (round edge thick rectangle), and 2, 3 and 4 (thick rectangle). Average *P2 *values are given besides the line denoting the position of the introgression. Dashed lines are used for IG males with average *P2 *not significantly higher than *D. mauritiana *males. The thinner dashed line is used for one IG line that is not significantly different than *D. mauritiana *only when the reduced data set is used for analysis (see results). Asteriks identified two IG lines for which data is lost when the reduced data set is used. One *P2 *value significantly higher than *D. mauritiana *(underlined) containing the two candidate loci for CSP is suggestive of the possible existence of suppressor somewhere between 73A10 and 77B map position. Two other introgressions (within region 5) outside the mapped loci with average *P2 *nonsignificantly different than *D. mauritiana *are suggestive of other loci responsible for second male paternity breakdown. An inversion (relative to *D. melanogaster *map) is shown in the X-axis.

A total of 2,635 *D. simulans ebony *females were set up to doubly mate, first with a male of the same strain then with a male from one of the 60 different IG lines. Females were removed from the final analysis if they failed to mate with either the first or the second male. Under these criteria, 913 females were excluded from the analysis leaving us with a total sample of 1,722 females. The proportion of progeny sired by the second (IG) male (*P2*) was angular transformed (*TP2*) to better fit the assumption of a normal distribution required for ANOVA. The second male paternity success scores were positively and significantly correlated with measures of female induced fecundity (Pearson correlation: *R*= 0.119; *P *< 0.001) so we tested variation among the average *P2 *scores of different IG lines using fecundity as a covariate. We found significant variation in *P2 *scores among IG lines (F_59,1599 _= 7.11; P < 0.001) with twelve IG lines having *P2 *scores not significantly higher than *D. mauritiana *males (t-test IG *vs*. *D. mauritiana *with *P *< 005) (Figure 1). We also found a significant block effect (F_8,1599 _= 5.71; P < 0.001) suggesting that part of the differences detected between IG males could be due to variation in environmental conditions among block trials. However, we found no significant male line × block interaction (F_54,1599 _= 1.15; P = 0.219) showing consistent scores of IG lines averages over blocks. The data were reanalyzed using only IG lines for which at least ten males successfully mated and excluding females that produced fewer than twenty offspring [34]. Under these criteria, we found consistent results of significant variation among IG lines (F_52,1249 _= 9.33; P < 0.001) and a significant block effect (F_8,1249 _= 4.89; P < 0.001) but a non-significant male line × block interaction (F_44,1249 _= 1.26; P = 0.125). Under these conditions, eleven IG lines have *P2 *scores not significantly higher than *D. mauritiana *males. Ten of them are among the twelve previously identified IG lines, the data restriction led to no data from two lines of the original twelve, and one IG line is added to the previous list as not significantly different from *D. mauritiana *males (Figure 2).

We found that all unhatched eggs were unfertilized indicating that the *P2 *values are not affected by differential zygote viability among strains. Another variable likely to affect sperm competitive ability is copulation duration. We assayed copulation duration from a subset of males from 12 IG strains with different average *P2 *scores and found no variation among strains (F_11,188 _= 1.13; P = 0.341), with all strains showing average copulation duration times more similar to *D. simulans *than to *D. mauritiana *males.

Figure 2 shows the *D. mauritiana *introgressions as mapped in a previous study [36]. Five regions, numbered one to five and shown as consecutive white and grey areas, can be broadly defined in the map on the basis of the introgression locations. All introgressions that span over regions two, three and four cause average *P2 *scores not significantly higher than *D. mauritiana *males (Figure 2, thick rectangle). This suggests that loci causing breakdown on second male paternity success must be located within these three regions. However, the fact that introgressions spanning regions two and three or three and four do not cause a breakdown in *P2 *(Figure 2, circle) suggests that all three loci defined by regions two, three and four, or at least two loci defined by regions two and four, cause second male paternity success breakdown. A size effect or the possibility of other loci contributing to the phenotypic breakdown are suggested by introgressions spanning regions one, two, three and four (Figure 2, thick rounded rectangle) and from IG lines with introgressions in region five having average *P2 *scores expected under CSP (Figure 2).

The two loci (Figure 2, regions two and four) that are at least needed to cause second male paternity breakdown correspond to map positions 77B to 84B and 88B to 92E as mapped by Tao and collaborators [36]. Within the mapped chromosome locations, we identified 81 broad-sense candidate genes on the basis of reproductive function and/or male reproductive tissue of expression (Additional file 1). It is important to note that even if we were to include a third locus (region three), the extension will not lead to additional candidate genes.

### A larger concentration of differentially expressed candidate genes is located in the 89B cytogenetic position

**Figure 3:**
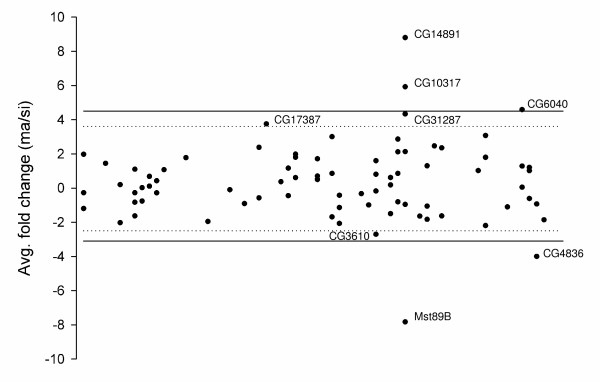
**Average fold difference in expression from male reproductive tract RNA extractions for 81 candidate genes between *D. simulans *and *D. mauritiana***. The differences in gene expression are shown as *D. mauritiana *relative to *D. simulans *(ma/si). The data is plotted with the X axis representing the cytogenetic map position. Experiment-wise statistical threshold at *P *< 0.05 and *P *< 0.1 are shown by solid and dotted lines respectively. Notice that 3 out of 5 genes showing significant differences in gene expression (*P *< 0.05) are located in map position 89B (CG14891, CG10317 and Mst89B).

We used 60 IG lines to map loci causing CSP, but we are ultimately interested in gene differences at mapped positions between pure species rather than IG lines. Therefore, differences in gene expression for all 81 broad-sense candidate genes were tested between *D. simulans *and *D. mauritiana*. We obtained RNA samples from the male reproductive tract of both *D. mauritiana *and *D. simulans *and performed quantitative real-time PCR (qRT-PCR) from reverse transcribed products corresponding to our 81 broad-sense candidate genes. We identified between five (CG10317, CG14891, Mst89B, CG6040 and CG4836) and eight (same as before plus CG3610, CG17387 and CG31287) candidate genes with significant differences in gene expression between the two species using either a five or ten percent threshold level respectively (Figure 3). This result is not qualitatively different when using two-fold average differences in gene expression and its 95% confidence interval as threshold (data not shown). Only one (CG17387) of the eight genes is located in the 77B to 84B. Three of the five differentially regulated genes (CG10397, CG14891 and Mst89B) as well as CG31287 are located in the 89B position suggesting that the evolution of species specific coregulation patterns of this gene cluster could be critical during species diversification and the evolution of CSP.

### DNA sequence data analysis of candidate genes

If species specific interactions are broken down in heterospecific crosses due to the presence of translated products that differ in function, we expect to see species specific signals in phylogenetic lineages leading to *D. simulans *and/or *D. mauritiana*. We tested all 81 broad-sense candidate genes for evidence of variation in rates of evolution among lineages in *D. melanogaster*, *D. simulans *and *D. sechellia *comparisons using currently available sequence data from the Drosophila species genome project [39]. Using a comparison of the free-ratios model to the one-ratio model of evolution available within PAML we identified eighteen genes, equally distributed along the two mapped loci as showing evidence of variable evolutionary rates among lineages. Six out of the 18 genes showed significant acceleration in the *D. simulans *lineage relative to the other two background lineages, with another three genes showing significant deceleration. One gene (CG14307 *fruitless*) showed both acceleration and deceleration, while CG31232 showed deceleration or no change, depending on the *D. melanogaster *alternative translation product used for analysis (Table 1 and Additional file 1). Particularly interesting are genes that demonstrate not only evidence of change in rates of evolution but also species specific adaptive diversification. Genes CG7478, CG31542, CG1984, CG3158, CG14307 and CG6255 exhibited both accelerated evolution and positive selection in the *D. simulans *lineage. Four other genes, CG9389, CG15179, CG31287 and CG4836, did not show evidence of a significant acceleration or deceleration but show evidence of positive selection in the *D. simulans *lineage (Table 1 and Additional file 1).

**Table 1:** Testing adaptive diversification in *D. simulans *using *D. melanogaster*, *D. simulans *and *D. sechellia *sequence comparisons.

Gene	Map	**ℓ**_**M0**_	**ℓ**_**M1**_	**2Δℓ**^**a**^	**ℓ**_**M2**_	**2Δℓ**^**b**^	ℓ(ω = 1)	ℓ(ω)	**2Δℓ**^**c**^
CG9936	78A	-12873.9	-12868.2	**11.5**	-12873.7	0.5	-12843.5	-12843.5	0.0
CG10510	78C	-2045.6	-2041.3	**8.5**	-2042.67	**5.9**	-2041.6	-2041.7	0.2
CG9389	78C	-2837.7	-2834.6	**6.2**	-2835.8	3.8	-2826.2	-2829.4	**6.3**
CG32436	78C	-7261.6	-7230.0	**63.2**	-7259.4	**4.5**	-7218.2	-7218.2	0.0
CG7405	78F	-1422.1	-1415.9	**12.4**	-1421.7	0.8	-1422.1	-1422.1	0.0
CG7478	79A	-1288.0	-1283.4	**9.1**	-1283.4	**9.1**	-1276.9	-1281.2	**8.5**
CG31542	83A	-898. 2	-892.0	**12.3**	-893.4	**9.6**	-873.4	-887.2	**27.6**
CG1041	83E	-2402.3	-2394.8	**15.1**	-2399.3	**5.9**	-2386.2	-2382.2	0.0
CG15179	84A	-890.0	-889.2	1.7	NA	NA	-878.3	-888.5	**20.5**
CG1030	84A	-1954.5	-1946.0	**16.9**	-1953.7	1.6	-1936.7	-1936.7	0.0
CG1984	84B	-2081.0	-2074.0	**14.0**	-2074.3	**13.3**	-1992.6	-2014.7	**44.2**
CG7362^d^	88D	-2209.9	-2209.1	1.4	NA	NA	-2199.2	-2202.4	**6.3**
CG6125	88F	-3268.6	-3259.0	**19.2**	-3268.3	0.6	-3246.1	-3246.1	0.0
CG3158	89A	-4968.6	-4960.7	**15.7**	-4961.8	**13.6**	-4775.0	-4911.8	**273.6**
CG14891^d^	89B	-2687.7	-2685.4	4.7	NA	NA	-2688.1	-2691.4	**6.5**
CG31287	89B	-1220.8	-1220.5	0.6	NA	NA	-1203.8	-1216.2	**24.8**
CG6963	89B	-2061.3	-2057.8	**7.0**	-2060.1	2.5	-2053.4	-2053.4	0.0
CG31232^e^	91A	-664.5	-659.8	**9.5**	-661.7	**5.5**	-643.1	-643.1	0.0
CG14307^e^	91A	-4285.9	-4278.2	**15.4**	-4279.2	**13.4**	-4066.8	-4175.1	**216.5**
CG6255	92A	-2099.4	-2088.3	**22.1**	-2088.4	**21.9**	-1966.6	-2039.2	**145.3**
CG4836	92B	-6245.6	-6229.6	**32.1**	-6245.5	0.2	-6190.0	-6204.6	**29.1**
CG12249	92B	-3301.2	-3297.0	**8.4**	-3298.2	**6.0**	-3301.2	-3301.2	0.0

^a^The test statistics compares the likelihood (ℓ) of the free-ratios model (ℓ_M1_) to the likelihood of a model that assumes constant ratios along branches (ℓ_M0_). The test statistics follows a chi-square distribution with two degrees of freedom. Significant values (P < 0.05) are bolded.

^b^The test statistics compares the likelihood (ℓ) of a model allowing for an ω estimate along the *D. simulans *branch and a different ω estimate for the background branches (ℓ_M2_) to the likelihood of a model that assumes constant ratios along branches (ℓ_M0_). The test statistics follows a chi-square distribution with one degree of freedom. Significant values (P < 0.05) are bolded.

^c^The test statistics compares the likelihood (ℓ) of a branch-site model with the same model but fixing the foreground ω to 1. The test statistics follows a chi-square distribution with one degree of freedom. Significant values (P < 0.05) are bolded.

^d^Comparisons are *D. melanogaster *-- *D. simulans *-- *D. mauritiana*.

^e^Variable results depending on the *D. melanogaster *open reading frame used in the analysis (See Additional file 1).

We noticed that seven genes lacked *D. melanogaster *orthologs in *D. sechellia *and/or *D. simulans *due to the presence of indels and/or nucleotide changes leading to the occurrence of stop codons along the coding sequence (missing in *D. sechellia*: CG9391, CG34357, CG1041, CG7362, CG5178, CG14891; missing in *D. simulans*: CG9063, CG34357, CG7362). With the exception of CG34357, whose gene region spans 64 Kb (Flybase), we partially sequenced all other six gene coding sequences in *D. simulans *and/or *D. mauritiana *and found that the lack of orthology is either restricted to *D. sechellia *or simply the result of sequencing errors in the genome database entry (Additional file 2). We therefore used our *D. mauritiana*, and in some cases *D. simulans*, partial sequences to test for variable rates of evolution and positive selection along the *D. simulans *lineage in *D. melanogaster*, *D. simulans *and *D. mauritiana *sequence alignments. Genes CG7362 and CG14891 showed evidence of positive selection along the *D. simulans *branch (Table 1 and Additional file 1).

With the exception of CG14307, we also partially sequenced *D. mauritiana *for all 12 genes showing evidence of positive selection along the *D. simulans *lineage (Table 1) and tested them using both PAML branch and branch-site models using the *D. simulans*, *D. sechellia *or *D. melanogaster*, *D. mauritiana *trio. In the 77B-84B locus, we detected evidence of positive selection in either *D. simulans *and/or *D. mauritiana *for CG7478, CG31542, CG1984 and CG1041. In the 88B-92E locus, CG7362, CG3158, CG31287, CG14891, CG6255 and CG4836 all showed evidence of positive selection. Only two (CG31287 and CG14891) of these ten genes are located within a common cytogenetic map position, 89B (Table 2). This result reinforces our previous observation, based on gene expression analysis, that the 89B position might have been critical during species diversification and the evolution of species specific adaptations to postmating prezygotic reproductive challenges.

**Table 2:** Testing adaptive diversification in *D. simulans *and/or *D. mauritiana *using *D. mauritiana*, *D. simulans *and *D. sechellia *sequence comparisons.

		***D. simulans***^**a**^				***D. mauritiana***^**a**^			
**Gene**^b^	**Map**	**ℓ (ω = 1)**	**ℓ (ω)**	**2Δℓ**^**c**^	**ω**	**ℓ (ω = 1)**	**ℓ (ω)**	**2Δℓ**^**c**^	**ω**

CG9389	78C3	-2413.9	-2413.9	0		-2413.9	-2413.9	0	
CG7478	79A6	-1212.7	-1206.4	12.6***	713.4	-1213.8	-1213.8	0	
CG31542	83A1	-837.1	-822.5	29.2***	62.8	-837.3	-837.3	0	
CG1041^d^	83E4	-2386.2	-2382.2	0		-2382.2	-2341.1	82.2***	999
CG15179	84A1	-747.2	-747.2	0		-747.1	-747.1	0	
CG1984	84B2	-1861.6	-1841.2	40.8***	20.6	-1865.5	-1865.5	0	
CG7362^d^	88D2	-2202.4	-2199.2	6.4*	999	-2202.4	-2199.2	6.4*	999
CG3158	89A5	-4249.9	-4232.3	35.2***	999	-4250.2	-4250.2	0	
CG31287	89B7	-1041.3	-1021.5	39.6***	999	-1041.3	-1041.3	0	
CG14891^d^	89B20	-2691.4	-2688.1	6.5*	17.4	-2691.4	-2688.5	5.7*	15.7
CG6255	92A5	-1229.1	-1189.7	78.7***	999	-1230.1	-1230.1	0	
CG4836	92B4	-5881.8	-5881.8	0		-5880.8	-5864.5	32.6***	999

^a^Foreground branch being tested.

^b^Candidate genes previously detected as experiencing positive selection along the *D. simulans *branch in comparisons with *D. melanogaster *and *D. sechellia *or with *D. melanogaster *and *D. mauritiana *(see Table 1).

^c^For each gene tested, we compared the likelihood (ℓ) of the branch-site model (Model = 2; NSsite = 2) with the same model but fixing the ω value of the foreground branch to 1. The test statistics follows a chi-square distribution with one degree of freedom. *** P < 0.001; ** P < 0.01; *P < 0.05.

^d^Comparisons are *D. melanogaster *-- *D. simulans *-- *D. mauritiana*.

## Discussion

The use of third chromosome *D. mauritiana *introgressions within a *D. simulans *genomic background has allowed us to establish the effect of such introgressions on a male's ability to father progeny when second to mate. We found significant variation in second male paternity success between the IG lines tested and also a significant correlation between second male paternity success and female induced fecundity. This correlation is expected given that sperm competition measured as a proportion of the progeny sired by the tester male is a subset of his ability to stimulate female progeny production (i.e. fecundity) and that such correlation has been previously found in similar tests using Drosophila strains [29,34]. The correlation detected in our study raises the possibility that lower second male paternity success might be due to poor fertility of the IG males. This possibility is likely given that some of the IG strains used in our test have been reported to be subfertile or even sterile when the *D. mauritiana *introgression is in homozygote condition [36]. However, we found that differences in female induced fecundity by IG males are not responsible for low sperm competitive ability. Our result is in agreement with prior findings showing that *D. simulans *females singly inseminated by a conspecific male produce a significantly higher average number of progeny than *D. simulans *females singly mated with *D. mauritiana *males, but that such reduced female fecundity is not enough to explain CSP [32,33].

While the extent of variation in P2 among IG lines highlights the complex genetic basis of male reproductive success, we have identified a chromosome introgression responsible for CSP. Two regions within it appear to be co-required to breakdown male competitive paternity success. Moreover, while previous studies have established associations between single genes and variation in first and second male paternity success in *D. melanogaster *[28,29], this study is the first to establish such associations in crosses between closely related species of Drosophila. We therefore provide a genetic basis for a well characterized postmating prezygotic isolation barrier in Drosophila that has been elusive in an earlier quantitative trait loci study [34].

Our mapping result identifies a minimum of two loci but does not rule out additional loci that could cause and/or suppress the phenotype. The presence of suppressor loci is suggested by males of one IG line showing no breakdown in second male paternity despite the presence of the two causative loci (Figure 2, underlined *P2 *value). There are also two introgressions outside the mapped area that could be additional loci influencing second male paternity success. Our mapping indicates that multiple genetic elements and gene interactions likely underlie the genetic basis of conspecific sperm precedence in Drosophila. This observation is in line with the view that complex epistasis plays a major role during evolution, species differentiation, and isolation. The identification of an epistatic basis of conspecific sperm precedence does not necessarily rule out the existence of genes of larger effect within our two mapped loci. An example is that the original mapping of *Odysseus *as a locus causing hybrid male sterility [40] was followed by the identification of additional nearby genes needed to cause full hybrid male sterility [41]. Nevertheless, *Odysseus *is now known as a gene that normally functions in spermiogenesis by increasing sperm production in young males [42] and has become a clear example of how an introgression mapping approach can lead to the identification of single genetic elements that underlie interspecies isolation.

We have identified a series of candidate genes within the mapped loci on the basis of gene regulatory differentiation and changes in coding sequences driven by adaptive diversification between *D. simulans *and *D. mauritiana*. Five of the eight candidates identified on the basis of differential gene expression had been previously shown to be differentially expressed in *D. simulans *and *D. mauritiana *using microarray analysis of testes gene expression [43] and the other three are genes coding for sperm proteins [44]. An interesting observation is that four out of the eight differentially expressed genes are located within the 89B cytogenetic map position, and so are two of the ten genes showing evidence of positive selection in the *D. simulans *and/or *D. mauritiana *lineage. It is possible that selection on protein coding genes and coevolution with DNA binding regulatory elements in this particular mapped position could play a major role during the evolution of postmating prezygotic isolation barriers. Selection driven coevolution has been demonstrated for X chromosome dosage compensation and the misregulation of X-linked genes in Drosophila hybrids that can lead to inviability [45].

Ten candidate genes were identified on the basis of adaptive diversification along the *D. simulans *and/or *D. mauritiana *lineage. The information available for these genes from studies in *D. melanogaster *reveal little more than the fact that they are linked to male reproduction on the basis of their expression in testes. In species like Drosophila, where females multiply mate, it is logical to assume that the adaptive diversification detected for these reproductive genes might be driven by their role in competition for fertilization through male - female and/or male - male interactions. However, it is interesting to note that a few of these candidate reproductive genes possibly exert an effect through genetic conflict and pleiotropic effects. Mutations in one of our mapped candidate genes, CG3158 (*spnE*), disrupt Piwi interacting RNA (piRNA) formation and therefore increases the activity of retrotransposons [46,47]. Selfish genetic elements like transposons are known to manipulate sperm and to impair sperm competitive ability [48,49]. Two other candidate genes, CG7362 and CG7478, have been characterized as members of the phagocytosis innate immunity system [50,51] and previous studies have shown evidence of tradeoffs between immune function and male reproductive success [52,53].

Future studies will need to focus on both functional assays and evolutionary analysis of the genes highlighted in this study. For example, Mst89B protein has been suggested to interact with Cdlc2, a microtubule motor activity protein expressed in the sperm, as well as the transcription regulator Brinker (Brk) [37]. In turn, both Cdlc2 and Brk might interact with Acp62F, an accessory gland protein shown to increase a male's ability to place sperm in storage when the gene is knocked out by targeted deletions [38]. A population survey of sequence variation at Acp62F has also established significant associations between polymorphisms at this gene and both second male paternity success and female induced fecundity via a genetic interaction with another Acp [29]. Therefore, it will be important to establish whether a single knockdown of *Mst89B *in *D. simulans *is capable of disrupting second male paternity success. Other mapped candidate genes from this study would need to be selected for simultaneous knockdowns, with proper controls to single out spurious side effects due to impaired overall viability/fertility caused by the knockdown.

## Conclusions

We identified at least two loci responsible for conspecific sperm precedence. The power of the associations established in our study is in its capacity to narrow down, by testing the effect of a large number of genetically manipulated lines on phenotypic variation, a large number of genes to a manageable number of candidate genes. A third of the candidate genes located within these two loci, showing differential gene expression or signature of adaptive diversification between parental species, are located in the 89B map position. Our finding highlights a potential major role for this chromosome position during the evolution of species specific adaptations to postmating prezygotic reproductive challenges.

## Methods

### Fly Stocks and maintenance

We used males from a set of 60 *D. simulans *strains that contain *D. mauritiana *mapped introgression into their third chromosome (IG lines). Each generation, the IG lines were maintained by selecting orange eyed males carrying a *D. mauritiana *P-element insert and crossing them to virgin females from a *D. simulans *B strain (white eyes) [36]. A stock of *D. simulans ebony *mutant flies (*e*/*e*) (black body color) and a wild-type stock of *D. mauritiana *were acquired from the Drosophila Species Stock center (UC San Diego, California: *D. simulans *stock 14021-0251.033; *D. mauritiana *stock 14021-0241.01). All stocks were maintained in bottles containing standard cornmeal-molasses media on a 12 hour light/dark schedule at 22°C. Every generation, parental flies were collected and placed into new bottles, left to mate and adults dumped after seven days. Prior to setting up crosses for the experiments, males and females were collected from the stocks on a 5 hour cycle to ensure virginity. Collection and sexing of the flies was carried out under light CO_2 _gas anesthetic. Males and females were separately aged to 3-6 days old in cornmeal-molasses vials containing no more than 20 flies.

### Phenotypic assays

Virgin 3-6 day old *Drosophila simulans *females homozygous for the *ebony *(*e/e*) mutation were mated to same-aged *D. simulans ebony *males. The mating was done *en masse *for a period of two hours in a vial containing 10 females and 20 males. Females were then individually aspirated to separate vials (vial 1) and males were discarded. Two days later, each female was presented with two males, from an IG line, that were heterozygous (orange eyes) for the *D. mauritiana *introgression. Heterozygous were used because some homozygotes are subfertile or sterile [36]. Mating was observed every 15 minutes for a total period of 8 hours. Males were discarded and females were aspirated into new vials (vial 2). Four days later each female was individually transferred to vial 3. Progeny from vials 1, 2 and 3 were counted on the 23^rd ^day after the beginning of oviposition and scored based on phenotypic body coloration. Females that did not produce *ebony *progeny in vial 1 were discarded from further analysis (i.e. no first mating). The fraction of wild-type progeny in vials 2 and 3, sired by the IG male, was designated as *P2*. The 60 strains were tested over time in nine blocks with partial replicates. We also tested the sperm competitive ability of a *D. mauritiana *wild-type strain when second to mate. An analysis of variance (ANOVA) was conducted using an angular transformation of *P2 *scores for the 60 IG strains tested, with fecundity as a covariate and both strain and block included as factors. Males from an IG line showing an average *P2 *score not significantly higher than *D. mauritiana *males were considered as not fitting the expected second male sperm precedence pattern commonly observed in intraspecific tests of sperm competition. Because the *D. mauritiana *introgressions have been previously mapped [36] we were able to establish associations between mapped *D. mauritiana *introgressions and CSP.

Low average *P2 *scores could be influenced by copulation duration or viability differences among IG lines under non-competitive settings. We therefore tested males from a subset of 12 IG strains. Copulation between virgin 3-6 days old *D. simulans ebony *females and IG males was observed and timed. Virgin *D. simulans *(*e/e*) females aged 3-6 days were mated *en masse *in a vial containing cornmeal-molasses media to males from one of the IG lines. Ten females were placed in a vial with 15-20 males from an IG line and left together for a period of eight hours at the end of which time the females were individually transferred to egg-laying dishes and inspected daily so that eggs that failed to hatch could be checked for evidence of fertilization (i.e. cell division).

### Mapping loci an candidate genes

Candidate genes within mapped introgressions causing CSP were identified using the genetic map of *D. melanogaster *available at Flybase http://flybase.bio.indiana.edu/. Candidate genes were selected based on chromosome location and narrowed down by focusing on genes expressed in male reproductive tissue. Tissue of gene expression was determined using a gene expression search via term link available at Flybase. Termlink categorizes genes by anatomy followed by organ systems. Within organ system it was possible to narrow down the search to the male reproductive system. We also performed Gene Ontology searches via Term-Link using broad search terms associated with sex and reproduction. Additional candidate genes were identified, on the basis of mapped chromosome position, from the *Drosophila melanogaster *sperm proteome [44], from a study that examined differences in gene expression between closely related *Drosophila *sister species, and from candidate accessory gland proteins [43,54]. The IG lines were used as mapping tools, however; the ultimate goal of our study was to identify relevant gene changes between parental species within the mapped chromosome locations. Therefore, candidate genes were tested for gene expression and DNA sequence differences in comparisons using data from *D. simulans *and *D. mauritiana*.

### DNA sequence data analysis

The DNA sequences of candidate genes were retrieved from Flybase as well as genome alignment sequences data available for *D. melanogaster*, *D. simulans *and *D. sechellia *at the UCSC genome browser http://genome.ucsc.edu/. Amino acid sequence alignments were performed using the ClustalX program and the alignments were used to generate nucleotide sequence alignments using Pal2Nal [55]. We tested for significant variation in ω (d_N_/d_S _per codon) rates of evolution along branches leading to each of the species by comparing the likelihood of a free-ratios model of evolution (M1) to the likelihood of constant ratio of evolution (M0) using the PAML software package [56]. Genes showing significant variation in rates of evolution across lineages were further tested for evidence of acceleration and/or deceleration of evolutionary rates along the *D. simulans *lineage by comparing the likelihood of a model that allows to estimate different ω values for the foreground *D. simulans *branch and the other background branches (M2) to the likelihood of the constant (M0) ratio model. We also tested for evidence of positive selection along the *D. simulans *branch using the mixed branch-site model (model = 2; NSsites = 2) within codeml [57]. The log-likelihood of the branch-site model is compared to the same model but fixing the ω value of the foreground *D. simulans *branch to 1 so that any significant variation in ω between foreground and background branches can be attributed to positive selection as opposed to differences in selective constraints [58]. It is possible that fast evolution and positive selection might also occur along other branches in the Drosophila phylogeny. However, the purpose of our study is to identify genes as candidates for CSP between *D. simulans *and *D. mauritiana*. Here we limit our analysis to the *D. simulans *lineage in *D. melanogaster*, *D. simulans *and *D. sechellia *comparisons.

According to Flybase, some *D. melanogaster *genes lacked orthologs in *D. simulans *and/or *D. sechellia*. Genes lacking orthologs in *D. simulans *were partially sequenced in *D. simulans *(From A.G. Clark; strain 'sim2', Winters, CA) to confirm nucleotide changes and/or indels found in the published sequences (UCSC genome browser) that lead to stop codons. Genes for which orthologs are reported missing in *D. sechellia *were partially sequenced in *D. mauritiana *(Drosophila Species Stock Center, 14021-0241.01). We also sequenced *D. mauritiana *for genes showing evidence of positive selection suggesting *D. simulans *species specific adaptations. Oligonucleotide primers for PCR and sequencing were designed using Primer3 software [59] on the basis of conserved sequence regions in the *D. melanogaster*, *D simulans *and *D. sechellia *alignments. PCR products were cleaned using the Wizard SV gel and PCR Clean-up system kit (Promega) and sequenced on both strands using a Beckman Coulter CEQ 2000XL automated sequencer (primers and PCR conditions available upon request). All *D. mauritiana *and *D. simulans *sequences can be found in GenBank under accession numbers GU931390 to GU931405.

*D. mauritiana *and *D. simulans *sequences were tested for the presence of an open reading frame. The PAML analysis was repeated as described before but using the *D. simulans*, *D. mauritiana *and *D. sechellia *(or *D. melanogaster*) trio and testing for changes in rates of evolution as well as positive selection along the *D. simulans *and the *D. mauritiana *branches.

### Gene expression data analysis

Male reproductive tracts were dissected from virgin *D. simulans *and *D. mauritiana *aged 3-6 days old. Dissections were carried out in 20 μl of PBS using fine forceps under a dissecting light microscope. Each sample consisted of reproductive tracts from 50 males. The tissue was stored at -20°C in 400 μl of RNA *later*^® ^Tissue Collection: RNA Stabilization Solution (Ambion). Tissue was removed from RNA *later*^® ^following manufactures suggested protocols by pelleting tissue in equal volume of ice cold PBS. RNA was isolated from the stored tissue using a TRIzol based RNA extraction for tissue protocol. The RNA pellet was resuspended in 40 μl of RNAse and nuclease free water and stored at -70°C.

The relative transcript abundance of candidate genes was determined in *D. simulans *and *D. mauritiana *by performing Real-Time quantitative PCR (RT-qPCR) using the MiniOpticon Real-Time detection system from Bio-Rad. Oligonucleotide primers were designed using Primer3 software [59] and on the basis of conserved sequence regions in the *D. melanogaster*, *D simulans *and *D. sechellia *alignments. An iScript™ One-Step RT-PCR Kit with SYBR^® ^Green was used according to manufacturers suggested protocols with the only modifications being the use of a 12.5 μl reaction volume and a three step PCR reaction. Ct values were normalized to a reference gene (*RpL32*) for each species and then fold differences in relative expression were calculated between species using the 2^-ΔΔCt ^calculation [60]. Each gene was tested twice using biological replicates and an average fold difference was calculated. To test for significant differences in gene expression and to control for experiment wise type I errors for the large number of multiple tests, we generated an experiment wise statistical threshold by using the five and ten percent tails of a population of 1,000 average values obtained by randomly sampling from the data with replacement.

## Authors' contributions

LL carried out the phenotypic assays, some of the gene expression and sequencing data collection and analysis, and drafted the manuscript. VS contributed to complete the gene expression assays and BB to the DNA sequencing data collection. AC conceived and coordinated the study, helped with data analysis and to draft the manuscript. All authors read and approved the final manuscript.

## Supplementary Material

Additional file 1 **Results from Phylogenetic Analysis by Maximum Likelihood (PAML) analysis of candidate genes using *D. melanogaster*, *D. simulans *and *D. sechellia *gene sequence data from the Drosophila 12 Genomes Consortium **[39]. Click here for file

Additional file 2 **Test of orthology in *D. simulans *and *D. mauritiana*. Nucleotide alignments between *D. simulans *(Dsim) *D. sechellia *(Dsec) and *D. mauritiana *(Dmau) are shown.** Dmau and DsimL are sequences generated in our lab. Stop codons due to indels (see carat) or nucleotide changes are bolded and underlined. Full sequence data can be found under accession numbers GU931390 to GU931405. CG34357 spans approximately 64 Kb (Flybase) and was not sequenced. Click here for file
