# Supplementary material for: Third chromosome candidate genes for conspecific sperm precedence between D. simulans and D. mauritiana
Source: BMC Genet. 2010 Apr 13;11:21. doi: 10.1186/1471-2156-11-21 (PMC2864193; doi:10.1186/1471-2156-11-21)
Supplement: Additional file 2 — Test of orthology in D. simulans and D. mauritiana. Nucleotide alignments between D. simulans (Dsim) D. sechellia (Dsec) and D. mauritiana (Dmau) are shown. Dmau and DsimL are sequences generated in our lab. Stop codons due to indels (see carat) or nucleotide changes are bolded and underlined. Full sequence data can be found under accession numbers GU931390 to GU931405. CG34357 spans approximately 64 Kb (Flybase) and was not sequenced. [file 1471-2156-11-21-S2.DOC]

**Additional file 2**: Test of orthology in *D. simulans* and *D. mauritiana*. Nucleotide alignments between *D. simulans* (Dsim) *D. sechellia* (Dsec) and *D. mauritiana* (Dmau) are shown. Dmau and DsimL are sequences generated in our lab. Stop codons due to indels (see carat) or nucleotide changes are bolded and underlined. Full sequence data can be found under accession numbers GU931390 to GU931405. CG34357 spans approximately 64Kb (Flybase) and was not sequenced.

**CG5178**

Dsim ATGTGTGACGATGATGCGGGTGCATTAGTTATCGACAACGGATCGGGCATGTGCAAAGCC

Dmau ---------------------------------------------GGCATGTGCAAAGCC

Dsec ATGTGTGACGATGATGCGGGTGCATTAGTTATCGACAACGGATCGGGCATGTGCAAAGCC

Dsim GGCTTCGCCGGTGATGACGCTCCCCGTGCTGTCTTCCCCTCGATTGTGGGTCGTCCCCGT

Dmau GGCTTCGCCGGTGATGACGCTCCCCGTGCTGTCTTCCCCTCGATTGTGGGTCGTCCCCGT

Dsec GGCTTCGCCGGTGATGACGCTCCCCGTGCTGTCTTCCCCTCGATTGTGGGTCGTCCCCGT

Dsim CACCAGGGTGTGATGGTGGGCATGGGTCAGAAGGACTCGTACGTGGGCGACGAGGCGCAG

Dmau CACCAGGGTGTGATGGTGGGCATGGGTCAGAAGGAATCGTACGTGGGCGACGAGGCGCAG

Dsec CACCAGGGTGTGATGGTGGGCATGGGTCAGAAGGACTCGTACGTGGGCGACGAGGCGCAG

Dsim AGCAAGCGCGGTATCCTGACGCTGAAGTACCCCATCGAGCACGGCATCATCACGAACTGG

Dmau AGCAAGCGCGGTATCCTGACGCTGAAGTACCCCATCGAGCACGGCATCATCACGAACTGG

Dsec AGCAAGCGCGGTATCCTGACGCTGAAGTACCCCATCGAGCACGGTATCATCACGAACTGG

Dsim GACGACATGGAGAAGATCTGGCATCACACCTTCTACAACGAGCTGCGCGTGGCCCCCGAG

Dmau GACGACATGGAGAAGATCTGGCATCACACCTTCTACAACGAGCTGCGCGTGGCCCCCGAG

Dsec GACGATATGGAGAAGATCTGGCATCACACCTTTTACAACGAGCTGCGCGTGGCCCCCGAG

Dsim GAGCATCCAGTATTATTGACCGAGGCACCCCTGAACCCCAAGGCCAATCGCGAGAAGATG

Dmau GAGCATCCAGTATTATTGACCGAGGCACCCCTGAACCCCAAGGCCAATCGCGAGAAGATG

Dsec GAGCATCCAGTATTATTGACCGAGGCACCCCTGAACCCCAAGGCCAATCGCGAGAAGATG

Dsim ACCCAGATCATGTTCGAGACCTTCAACTCGCCGGCCATGTACGTGGCCATCCAGGCCGTG

Dmau ACCCAGATCATGTTCGAGACCTTCAACTCGCCGGCCATGTACGTGGCCATCCAGGCCGTG

Dsec ACCCAGATCATGTTCGAGACCTTCAACTCGCCGGCCATGTACGTGGCCATCCAGGCCGTG

Dsim CTCTCCC-TGTACGCCTCTGGTCGTACCACCGGTATTGTGCTGGACTCCGGCGATGGTGT

Dmau CTCTCCC-TGTACGCCTCCGGTCGTACCACCGGTATTGTGCTGGACTCCGGCGATGGTGT

Dsec CTCTCCCCTGTACGCCTCCGGTCGTACCACCGGTATTGTGCTGGACTCCGGCGATGGTGT

^

Dsim CTCCCACACCGTACCCATCTATGAGGGCTTCGCCCTGCCCCACGCCATCCTGCGTCTGGA

Dmau CTCCCACACCGTGCCCATCTATGAGGGCTTCGCCCTGCCCCACGCCATCCTGCGTCTGGA

Dsec CTCCCACACCGTGCCCATCTA**TGA**GGGCTTCGCCCTGCCCCACGCCATCCTTCGTCTGGA

Dsim TCTGGCTGGTCGCGATCTGACCGATTACCTGATGAAGATCCTGACGGAGCGCGGCTACAG

Dmau TCTGGCTGGTCGCGATCTGACCGATTACCTGATGAAGATCCTGACGGAGCGCGGCTACAG

Dsec TCTGGCTGGTCGCGATCTGACCGATTACCTGATGAAGATCCTGACGGAGCGCGGCTACAG

Dsim CTTCACCACCACCGCCGAGCGTGAGATCGTGCGCGACATCAAGGAGAAGCTGTGCTACGT

Dmau CTTCACCACCACCGCCGAGCGTGAGATCGTGCGCGACATTAAGGAGAAGCTGTGCTACGT

Dsec CTTCACCACCACCGCCGAGCGTGAGATCGTGCGCGACATCAAGGAGAAGCTGTGCTACGT

Dsim GGCTCTGGACTTCGAGCAGGAGATGGCCACCGCTGCCGCCTCCACCTCGCTGGAGAAGTC

Dmau GGCTCTGGACTTCGAGCAGGAGATGGCCACCGCTGCCGCCTCCACCTCGCTGGAGAAGTC

Dsec GGCTCTGGACTTCGAGCAGGAGATGGCCACCGCTGCCGCTTCCACCTCGCTGGAGAAGTC

Dsim GTACGAGTTGCCCGACGGCCAGGTGATCACCATTGGCAACGAGCGCTTCCGCTGCCCCGA

Dmau GTACGAGTTGCCCGACGGCCAGGTGATCACCATTGGCAACGAGCGCTTCCGCTGCCCCGA

Dsec GTACGAGTTGCCCGACGGCCAGGTGATCACCATTGGCAACGAGCGCTTCCGCTGCCCCGA

Dsim AGCCCTGTTCCAGCCCTCGTTCCTGGGCATGGAGTCGTGCGGCATCCACGAGACCGTCTA

Dmau GGCCCTGTTCCAGCCCTCGTTCCTGGGCATGGAGTCGTGCGGCATCCACGAGACCGTCTA

Dsec GGCCCTGTTCCAGCCCTCGTTCCTGGGCATGGAGTCGTGCGGCATCCACGAGACCGTCTA

Dsim CAACTCGATCATGAAGTGCGACGTGGACATCCGCAAGGATCTGTATGCCAACTCCGTGCT

Dmau CAACTCGATCATGAAGTGCGACGTGGACATCCGCAAGGATCTGTATGCCAACTCCGTGTT

Dsec CAACTCGATCATGAAGTGCGACGTGGACATCCGCAAGGATCTGTATGCCAACTCCGTGCT

Dsim GTCCGGCGGTACCACCATGTACCCTGGTATTGCCGATCGTATGCAAAAGGAGATCACTGC

Dmau GTCCGGCGGTACCACCANGTACCCTGGT--------------------------------

Dsec GTCCGGCGGTACCACCATGTACCCTGGTATTGCCGATCGTATGCAGAAGGAGATCACTGC

**CG9391**

Dsim ATGTCACACAGCGTGGACGTGGAAAAGTGCTTAGAGGTGGCCAGCAACCTGGTTTCAGAA

Dmau ATGTCACANNNCGTGGACGTGGAAAAGTGCTTAGAGGTGGCCAGCAACCTGGTTTCAGAA

Dsec ATGTCACACAGCGTGGACGTGGAGAAGTGCTTAGAGGTGGCCAGCAACCTGGTTTCAGAA

Dsim GCTGGAAGGCTCATCGCTCGCAACAATGAGCAGCGACAGGACTTCGTTTGCAAGAGCAAT

Dmau GCTGGAAGGCTCATCGCTCGCAACAATGAGCAGCGACAGGACTTCGTTTGCAAGAGCAAT

Dsec GCTGGAAGGCTCATCGCTCGCAACAATGAGCAGCGACAGGACTTCGTTTGCAAGAGCAAT

Dsim GACATCGACTTGGTGACCCAAACAGACAAGGATGTGGAGCAGCTACTGATGGACGGCATT

Dmau GACATCGACTTGGTGACCCAAACAGACAAGGATGTGGAGCAGCTACTGATGGACGGCATT

Dsec GACATCGACTTGGTGACCCAAACAGACAAGGATGTGGAGCAGCTACTGATGGACGGCATT

Dsim CGCCGCCACTTTCCGGAGCACAAGTTCATCGGCGAGGAGGAGAGTAGCGGCGAGGAGGGT

Dmau CGCCGCCACTTTCCGGAGCACAAGTTCATCGGCGAGGAGGAGAGTAGCGGCGGGGAGGGT

Dsec CGCCGCCACTTTCCGGAGCACAAGTTCATCGGCGAGGAGGAGAGTAGCGGCGAGGAGGGT

Dsim GTCAAGAAGCTTACCGACGA-GCCCACCTGGATCATTGATCCCGTGGACGGCACCATGAA

Dmau GTCAAGAAGCTTACCGACGA-GCCCACCTGGATCATTGATCCCGTGGACGGCACCATGAA

Dsec GTCAAGAAGCTTACCGACGAAGCCCACCTGAATCAT**TGA**TCCCGTGGACGGCACCATGAA

^

Dsim CTTTGTGCACGCATTTCCGCACTCTTGCATCT-CCGTGGGTCTGAAGGTGAACAAGGTCA

Dmau CTTTGTGCACGCATTTCCGCACTCTTGCATCT-CCGTGGGTCTGAAGGTGAACAAGGTCA

Dsec CTTTGAGCACGCATTTCCGCACTCTTGCATCTTCCGTGGGTCTGAAGGTGAACAAGGTCA

Dsim CGGAGCTGGGCTTGGTCTACAATCCCATCCTGGAGCAGCGCTTCACTGCGCGACGTGGGC

Dmau CGGAGCTGGGCTTGGTATACAATCCCATCCTGGAGCAGCGCTTCACTGCGCGACGTGGGC

Dsec CGGAGCTGGGCTTTGTCTACAATCCCATCCTGGAGCAGCGCTTCACTGCGCGACGAGGGC

Dsim ACGGAGCCTTCTACAACGGGCGCAGGATCCACGTGAGCGGCCAAAAGGAACTGGG-CAAA

Dmau ACGGAGCCTTCTACAACGGGCGCAGGATCCACGTGAGCGGCCAAAAGGAACTGGG-CAAA

Dsec ACGGAGCCTTCTACAACGGGCGCAGGATCCACGTGAGCGGCCAAAAGGAACTGGGGCAAA

Dsim GCGCTGGTCACCAGTGAATTCGGTACCACCCGGGACGAGGCCAAGATGAAGGTCGTGCAT

Dmau GCGCTGGTCACCAGTGAATTCGGTACCACCCGGGACGAGGCCAAGATGAAGGTCGTGCAT

Dsec GCGCTGGTCACCAGTGAATTCGGTACCACCCGGGACGAGGCCAAGATGAAGGTCGTGCAT

Dsim GAGAACTTCGAGAAGATGGCCAAAAAGGCGCATGGCCTACGGGTCCTGGGTTCGGCAGCC

Dmau GAGAACTTCGAGAAGATGGCCAAAAAGGCGCATGGCCTACGGGTCCTGGGTTCGGCAGCC

Dsec GAGAACTTCGAGAAGATGGCCAAAAAGGCGCATGGCCTACGGGTCCTGGGTTCGGCAGCC

Dsim CTTAATATGTCGATGGTTGCTCTGGGAGCCGCTGACGCCAACTACGAATTTGGAATTCAC

Dmau CTTAATATGTCGATGGTTGCTCTGGGAGCCGCTGACGCCAACTACGAATTTGGAATTCAC

Dsec CTTAATATGTCGATGGTTGCTCTGGGAGCCGCTGATGCCAACTACGAATTTGGAATTCAC

Dsim GCCTGGGATGTGTGTGCCGGCGACTTGATTGTCCGGGAGGCTGGTGGCGTAGTCATCGAT

Dmau GCCTGGGATGTGTGTGCCGGCGACTTGATTGTCCGGGAGGCTGGTGGCGTGGTCATCGAT

Dsec GCCTGGGATGTGTGTGCCGGCGACTTGATTGTCCGGGAGGCTGGTGGCGTGGTCATCGAT

Dsim CCTGCTGGCGGCGAATTCGACATCATGTCTCGAAGGGTCCTGGCGGCAGCCACACCAGAG

Dmau CCTGCTGGCGGCGAATTCGACATCATGTCTCGTAGGGTCCNGGCGGCN------------

Dsec CCTGCTGGCGGCGAATTCGACATCATGTCTCGAAGGGTCCTGGCGGCAGCCACACCAGAG

**CG14891**

Dsim ATGTTCCGCTTGAAGGGAAATTTTGGAAATTTTTACTTTAAAAATGGTTTGGTGTACACC

Dmau ------------------------------------------------------------

Dsec ATGTTCCGCTTGGGGGAAAATTTTGGAAATTTTTACTTTGAAAATGGTTTGGCGTACACC

Dsim AAAGATCGCAAGGTTGTGCGACTGGTGACGATCTCTGCGAACTGGCACTTTTCGAAGAGC

Dmau ---------AAGGTTGTGCGACTGGTGACGATCTCTGCGAACTGGCACTTTTCGAAGAGC

Dsec GAAGATCGCAAGGTTGTGCGACTGGTGACGATCTCTGCGAAGTGGCACTTTTCGAAGAGC

Dsim CAACTCTGGAAGCACTTCTCGAGTTTTGGAACTGTGGAGGATCTCCAATGGGAAAA-GGA

Dmau CAACTCTGGAAGCACTTCTCGAGTTTTGGAACTGTGGAGGATCTCCAATGGAAAAA-GAA

Dsec CAACTCTGGAAGCACTTCTCGAGTTTTGGAACTGTGGAGGATCTCCAA**TGA**AAAAAAGAA

^

Dsim TAAGAGAGTGGGATCGGTTCTTTTTCAAGAGGCTTCCCAAGCGGCAAAGGTTTTGGTGTT

Dmau TAAGAGAGTGGGATCGGTTCTTTTTCAAGAGGCTTCCCAAGCGGCAAGGGGTTTGGTGTT

Dsec **TAA**GAGAGTGGGATCGGTTCTTTTTCAAGAGGCTTCCCAAGCGGCAAGGGTTTTGGTGTT

Dsim GACTAAACACCATTTGTATGGCCATGTTCTTTATTTGCAGCCCAGCACCTCCAGGCGCGA

Dmau GGCTAAACACTATTTGTATGGCCATGTTCTATATTTGCAGCCCAGAACCTCCGGGCACGA

Dsec GACTAAACACTATTTGTATGGCCATGTTCTTTATTTGCAGCCCAGCACCTCCAGGCGCGA

Dsim ACCGCCGGTGAAGGAATCAGAAACTATTTCTGCCTACGATATACCCGTTGTCGATGACTT

Dmau ACCGCCGGTGAAGGAATCAGGAACTCTTTCTGCCTACGATATACCCGTTGTCGATGACGT

Dsec ACCGCCGGTGAAGGAATCAGAAACTATTTCTGCCTACGATACACCCGTTGTCGATGACGT

Dsim TTGGTATAAAGTGCTCGAATATCTTCCACTAAATGCCCGTCTCAACTTTGCCGCCAGTTG

Dmau TTGGTATAAAGTGCTCGAATATCTTCCACTAAATTCCCGTCTCAACTTTTCCGCCAGTTG

Dsec TTGGTATAAAGTGCTCGAATATCTTCCACTAAAATCCCGTCTCAACTTTGCCGCCAGTTG

Dsim TAAAAGATTCAAGACGATCTACGAATTGGAGTCGCGTCGTAACAATCGTGTTCTTAATAT

Dmau TAAAAGATTCAAGACGATCTACGAATTGGAGTCGCATCGTAACAATCGTGTTCTTAATAT

Dsec TAAAAGATTCAAGACAATCTACGAATTGGAGTCGCATCGTAACAATCGTGTTCTTAATAT

Dsim GAAGGATGTTTGCACACTGGACGACTTTGGCATTAAAATATTGATGCGGCTATCAGGAAA

Dmau GAAT---ATTTGCACACTGGACGACTTTGGCATTAAAATATTGATGCGGCTATCAGGAAA

Dsec GAGGGATGTTTGCACACTGGACGACT-------------------GCGGCTATCCGGAAA

Dsim ACACATTCATTGTGTAAAAGGTGGCCCGCTTCATTGGACGCTTATGTTGGAGTTCGTGCA

Dmau ACACATTCATTGTGTAAAAGGTGGCCCGCTTCATCGGCCGCTTATGTCGGAGTTCGTGCA

Dsec ACACATTCATTGTGTAAAAGGTGGCCCGCTTCATCGGCCGCTTATGTCGGAGTTCGTGCA

Dsim GCTATTGGGTGTAAGCTGTCCAAATCTAGCAGAGCTAAGTTTCTACAAAA--------TT

Dmau GCTATTGGGTGTAAGCTGTCCAAATCTAGCAGAGCTAAGTTTCTACAATA--------CT

Dsec GCTATTGGGTGTAAGCTGTCCAAATCTAGCAGAGCTAAGTTTCTACAATAGAGCTAAGTT

Dsim TCAGTCAGCCTAGACCACATGACTCACCTGTTCGATGGTGCCAATGGCTTGAATAATATC

Dmau TCAGTCAAC---------ATGACTCACCTGTTCGATGGTGCCAATGGCTTGAATAATATC

Dsec TCAGTCAGCCTAGACCACATGACTCACCTGTTTGATGGTGCCAATGGCTTAAATAATATC

Dsim ACCACCATATCCTTGAGGTGTTGTGACTTGGCAGATCCTCAAATTTACTGCTTGCAGATG

Dmau ACCACCATATCCTTGAGGTGTTGTGACTTGGCAGATACTCAAATTTACTGCTTGCAGATG

Dsec ACCACCATATCCTTGAGGTGTTGTGACTTGGCAGATACTCACATTTACTGCTTGCAGATG

Dsim CTATCTAAACTAAAGAGTCTGGACATCGCACAGAACCATTTCATTAGGGGCGAAAGTTTA

Dmau CTATCTAAACTAAAGAGTCTGGACATCGCACAGAACCATTTCATTAGGGGCGAAAGTTTA

Dsec CTATCTAAACTAAAGAGTCTGGACATCGTACAGAACTATTTCATTAAGGGCGAAAGTTTA

Dsim AACTCTCTGCCAATTTCCTTGGAGATTTTAAATGTTTCAAAATGCGACAGACTGCGGCCC

Dmau AACTCTCTGCCAATTTCCTTGGAGATTTTAAATGTTTCAAAATGCGATAGTCTGTTGCCC

Dsec AAATCTCTGCCAATTTCCTTGGAGATTTTAAATGTTTCAAAATGCGACAGACTGCTGCCC

Dsim AAGAATCTTATCAATCTTGCGTCCCTGACGCATCTCCGCGAACTGCGCTGCTCTGGCATT

Dmau AAGCATCTTATCAATCTTGCGTCCCTGTCGCATCTCCGCGAACAG---------------

Dsec AAGCATCTTATCAATCTTGCGTCCCTGTCGCATCTCCGCGAACTGCGCTGATCTGGCACT

Dsim TCCAAGCTTACGAAAAATGAGCTGTTCAAACGGTTCGCACATTACTGTCCAATGCTCGAG

Dmau ------------------------TACAAACGGTTCGCACATTACAGTCCAATGCTCGAG

Dsec TCCAAGTGTGCGAAAAATGAGCTGTACAAACGGTTCGCACATTACTGTCCAATGCTCGAG

Dsim GTTCTCGAGGTTACCGACATTATGAAGAAGATACAGCTGGGCGGTCTGTCTCGTCTCCAC

Dmau GTT---------ACCGACACTATGAAGAAGATACAGCTGGGCGGTCTGTCTCGTCTCCAC

Dsec GTTCTCGAGGTTACCGACATTATGCAGAAGATACAGCTGGGCGGTCTGTCTCGTCTCCAC

Dsim ACCTTGGTCATTCAGTCTTCCGAAGGGTCTGGCGACCATATGAATAACTTGATGCTTTCG

Dmau ACCTTGGTCATTCAGTCTTCCGAAGGGTTTGGCGACCATATGAATAACTTGCTGCTTACG

Dsec ACCTTGGTCATTCCGTCTGCCGAAGGGTTTGGCGACCATATGAATAACTTGCTGCTTACG

Dsim TCGATCGCGGAATCGTATTCGCTGCGCCGTCTGGAGATTATAGATTCTTTTGAACGTTTT

Dmau TCGATCGCGGAATCGTATTCGCTGCGCCATCTGGAGATTATAGATTCTTTTGAACGTTTT

Dsec TCGATCGCGGAATCGTATTCGCTGCGCCATCTGGAGATTATAGATTCTT-----------

Dsim TTCACTATTTCCTTCGATCTGAGTATTTTATCCCCGCTTAAAGAACTGCGGACCCTAATA

Dmau TTAGCTATTTCCTTCGATCTGAGTATTTTATCCCCGCCTAAAGAACTGCGGACCCTAATA

Dsec -----------------------------AACTTAACTTAAAGAACTGCGGACCCTAATA

Dsim TTACATAATCTGAACTTTACACCGGAACACCTAATGGGATTGCAAAAACTCCCTGCCTTG

Dmau TTACATAATCTGAACTTTACACCGGTACACCTAATGGGATTGCAAAAACTCACTGCCTTG

Dsec TTACATAATCTAAACTTTACACCGGTACACCTAATGGGATTGCAAAAATTCACTGCCTTG

Dsim GAGTTTCTGGACCTGAGTGGCTCGCCCGATCTATCCAATGAGGACGTTGCAAAGTTGACG

Dmau GAGTTTCTGGACCTGACTGGCTNGCCCGATCTATCCAATGAGGACGTTGCGAAGTTGACG

Dsec GAGTTTCTGGACCTGACTGGCTCGCGCGATCTATCCAATGAGAACGTTGCGAAGTTGACG

Dsim AAACCGCTGGGCAGACTGCGCCGACTAACGGTTGAGCGTTGTCCTTTTATCTCACGACAA

Dmau AAGCCGCTGGGCAGACTG------------------------------------------

Dsec AAACCGCTTGGCAGACT-----------------------------------CACGACAA

**CG9063**

Dsim TGGACAGTGTTGCAGCTGCCGCTCAACTACGCGGCGACAAACTGGCCAATCCGGTATGCT

Dsec TGGACAGTGTTGCAGCTGCCGCTCAACTACGCGGCGACAAACTGGCCAATCCGGTATGCT

DsimL --GACAGTGTTGCAGCTGCCGCTCAACTACGCGGCGACAAACTGGCCAATCCGGTATGCT

Dsim GCTATTGATCCGGATGGACTCCACTTGGCGGTGGCTGGTCGCACTGGGCTGGCGCACTAT

Dsec GCTATTGATCCGGATGGACTCCACTTGGCGGTGGCTGGTCGCACTGGGCTGGCGCACTAT

DsimL GCTATTGATCCGGATGGACTCCACTTGGCGGTGGCTGGTCGCACTGGGCTGGCGCACTAT

Dsim TCCCTAGTGACC-GGCGCTGGAAGCTTTT-GGCAA**TGA**GTCGCAGGAGAAGGACTTTGTT

Dsec TCCCTAGTGACCCGGCGCTGGAAGCTTTTTGGCAATGAGTCGCAGGAGAAGGACTTCGTT

DsimL TCCCTAGTGACCCGGCGCTGGAAGCTTTTTGGCAATGAGTCGCAGGAGAAGGACT-----

^ ^

**CG1041**

Dmau ------------------------------------------------------------

Dsec ---------------ATGTACAAATATCAAAGCCCAACGTCTGAGCGAATCCTCAAGAAA

Dsim ATGTTAATTAACCGGAGCAAAACAAGAGTGAGCCCAACGTCTGAGCGAATCCTCGAGAAA

Dmau ------------------------------------------------------------

Dsec CCCGACGCCGAGATGAAGTTTCGTGGCAATGGAAAGCTTTTGTGGAATTTGACCAAGAAC

Dsim CCCGACGCCGAGATGAAGTTCCGTGGCAATGGAAAGCTTTTGTGGAATTTGACCAAGAAC

Dmau ------------------------------------------------------------

Dsec TCCTTGGCCCAACAGTCTCCAAATGGGATCGCCAAGAAAGTGCTGCCCGCCAGCAGCTAC

Dsim TCCTTGGCCCAGCAGTCTCCAAATGGAATCGCCAAGAAAGTGCTGCCCGCCAGCAGCTAC

Dmau ---------------------------------------CTGCTGAAGTACCACGTCCTG

Dsec AGCACCGTCCAGAAGACCATTCCCTTGGAGCAGCCGAATCTGCTGAAGTACCACGTCCTG

Dsim AGCACCGTCCAGAAGACCATTCCCTTGGAGCAGCCGAATCTGCTGAAGTACCACGTCCTG

Dmau CCGCTGGAGGAAACGCTAAACCGCTTCATGACCACGGTGGAACCTCTGCTGACGCCGGAG

Dsec CCGCTGGAGGAAACGCTGAACCGCTTCATGACCACGGTGGAACCGCTGCTGACGCCGGAG

Dsim CCGCTGGAGGAAACGCTGAACCGCTTCATGACCACGGTGGAACCTCTGCTGACGCCGGAG

Dmau GAGTTTCAACAGCAAAAGGGAATCACCTCCGAGTTTTTGAAGAAGCAGGGACGCGAACTG

Dsec GAGTTTCAACAGCAAAAGGGAATCACCTCCGAGTTTTTGAAGAAGCAGGGACGCGAACTG

Dsim GAGTTTCAACAACAAAAGGGAATCACCTCCGAGTTTTTGAAGAAGCAGGGACGCGAACTG

Dmau CAGCTGCTCCTGGAAGAAACCGGCAGCAAGGAGAAGAATTGGCTGGCCCACCGCTGGCTG

Dsec CAGCTGCTCCTGGAAGAAACCGGCAGCAAGGAGAAGAATTGGCTGGCCCACCGCTGGCTG

Dsim CAGCTGCTCCTGGAAGAAACCGGCAGCAAGGAGAAGAATTGGCTGGCCCACCGCTGGCTG

Dmau AAGGCTGCCTATTTGACCTATCGAGACCCAGTCACCGTGTTCGTGAGTCCTGGCATGACC

Dsec AAGGCTGCCTATTTGACCTATCGAGACCCAGTCACCGTGTTCGTGAGTCCCGGCATGACC

Dsim AAGGCTGCCTATTTGACCTACCGAGACCCAGTCACCGTGTTCGTGAGTCCCGGCATGACC

Dmau TTCCCCAAGCAAAACTTCAGGGACTCACGCGCTTTCGTGGACTATACCGCCAGGGTTATC

Dsec TTCCCCAAGCAAAACTTCAGGGACTCACGGGCTTTCGTGGACTATACCGCGAGGGTTATC

Dsim TTTCCCAAGCAAAACTTCAGGGACTCACGCGCTTTCGTGGACTATACCGCCAGGGTTATA

Dmau TATGGCCTGGGCGANNTCAACGACATGGTGCACGCCAACCAAATTCCGATCGTTAAAATG

Dsec TATGGACTGGGCGAATTTAACGACATGGTGCACGCCAACCAAATTCCGATCGTTAAAATG

Dsim TATGGCCTGGGCGAATTCAACGACATGGTGCACGCCAACCAAATTCCGATCGTTAAAATG

Dmau GGCAAGAACGAGCGGGACAACAGCCAGTTTGGCAANGTATTCGGCACATGTCGGATT-CC

Dsec GGCAAGAACGAGCTGGACAACAGCCAGTTTGGCAAGGTATTCGGCACATGTCGGATTTCT

Dsim GGCAAGAACGAGCTGGACAACAGCCAGTTTGGCAAGGTATTCGGCACATGTCGGATT-CC

^

Dmau CAGACGGGACACCGACGAGATCGTATACAATCCCGACTCCGATTATGTGGTGGTGATCTA

Dsec CAGACGGGGCACCGACGAGATCGTATACAATCC**TGA**CTCCGATTATTTGGTGGTGATCTA

Dsim CAGACGGGGCACCGACGAGATCGTATACAATCCCGACTCCGATTATGTGGTGGTGATCTA

Dmau CAAGAATCACTTCTACCAACTGAAGATATACAGTAAGGAGGGAAAGCTCATTGCTGCTCC

Dsec CAAGAATCACTTCTACCAACTGAAGATATACAGTAAGGAGGGAAAGCTCATTGCTGCTCC

Dsim TAAGAATCACTTCTACCAACTGAAGATATACA----------------------------

Dmau ATGTCTAGCTGCTCAACTCGAGAATATCTTGTTGAAGGAAACGCAAGTGGGAGTACCTTA

Dsec ATGTCTAGCTGCTCAACTGGAGAATATCTTGTTGAAGGAAACGCAAGTGGGAGTACCCTA

Dsim --------CTGCTCAACTGGAGAATATCTTGTTGAATGAAACGCAAGTGGGAGTACCCTA

Dmau TGGTATTCTGACCACCGACTCCAGGGACANTTGGGCCCAACCCTACGAGTATCTGGCTGA

Dsec TGGTATTCTGACCACCGACTCCAGGGACAATTGGGCCGAAGCCTACGAATATCTGGCTGA

Dsim TGGTATTCTGACCACCGACTCCAGGGACAATTGGGCCGAAGCCTACGAATATCTGGCTGA

Dmau GACTCCTGGCAACCGGGATGCCCTCAAGACCATACAGAGTGCTCTGTTCACCGTCTCACT

Dsec AACTCCTGCCAACCGGGATGCCCTCAAGACCATACAGAGTGCTTTGTTCACCGTCTCACT

Dsim GACTCCTGGTAACCGGGATGCCCTCAAGACCATTCAGAGTGCTCTGTTCACCGTCTCACT

Dmau CGATGAGGGTACTAGCCTAAAGGACGGCGAAGAGACTGACGAACTTATTCTATCGCTGAT

Dsec CGATGAGGGTACTAGCCTAAAGGACGGCGAAGAGACTGACGAACTTATTCTATCGCTGAT

Dsim CGATGAGGGTACTAGCCTAAAGGAAGGCGAAGAGACCGACGAGATTATTCTATCGCTGAT

Dmau CCATGGCAGTGGCAGCAAGAGGAACAGCGGCAACCGCTGGATGGACAAGACTATTCAGCT

Dsec CCATGGCAGTGGCAGCAAGAGGAACAGCGGCAACCGTTGGATGGACAAGACTATTCAGCT

Dsim CCATGGCAGCGGCAGCAAGAGGAACAGTGGCAACCGCTGGATGGACAAGACTATTCAGCT

Dmau GGTGGTAAACCCCAATGGAAACGTCGGATTCACCTATGAGCACTCGCCGGCTGAGGGCCA

Dsec GGTGGTTAACCCCAATGGAAACGTCGGATTCACCTATGAGCACTCGCCGGCTGAGGGCCA

Dsim GGTGGTAAACCCCAATGGAAACGTCGGGTTCACCTATGAGCACTCGCCGGCTGAGGGCCA

Dmau GCCCATTGCGATGATGATGGACTACGTGGTGCAAAAGATGTANGAAGACCCTAGCTTCGG

Dsec GCCCATTGCGATGATGATGGACTACGTGGTGCAAAAGATGAAGGAAGACCCTAGCTTCGG

Dsim GCCCATTGCGATGATGATGGACTACGTGGTGCAAAAGATGAAGGAAGACCCTAGCTTCGG

Dmau GCAAAC-GGCTCACAGAACTT-GCTCCCGCACAGAAAATTCAGTTNNTCTCGAGCA-TAA

Dsec GCAAACTGGCTCACAGGACTTTGCTCCCGCACAGAAAATACAGTTCTCTTCGAGTAATAA

Dsim GCAAACTGGCTCACAGGACTTTGCTCCCGCACAGAAAATTCAGTTCTCTTCGAGCAATAA

Dmau AAGTCTAGAGAAATCTTTAAACGTGTCCCNGGCAANCGTGGACAAACTTGCCGATGCTCT

Dsec AAGTCTAGAGAAATCTTTAAACGTCGCACAGGCAAACGTGGACAAACTTGCCGATGCTCT

Dsim AAGTCTAGAGAAATCTTTAAACGTCGCCCAGGCAAACGTGGACAAACTTGCCGATGCTCT

Dmau CCAAATGAAGGTTCTGAAATTCACCGGCTTCGGAAAGGATTTCATAAAGAAACAGCGTCT

Dsec CCAAATGAAGGTTCTGAAATTCAACGGCTTCGGAAAGGATTTCATAAAGAAACAGCGTCT

Dsim CCAAATGAAGGTTCTGAAATTCACCGGCTTCGGAAAGGATTTCATAAAGAAACAGCGTCT

Dmau GGGTCCGGACAGCTTTGTTCAGATGGCTCTTCAGCTCGCCTTCTACAAAATGCACTCGGA

Dsec GGGTCCGGACAGCTTTGTTCAGATGGCGCTGCAGCTCGCCTTCTACAAAATGCACTCGGA

Dsim GGGTCCGGACAGCTTTGTTCAGATGGCGCTGCAGCTCGCCTTCTACAAAATGCACTCGGA

Dmau ACCGCCGGCGCAATATGAGTCGGCTCATCTGCGCATATTCGACGGTGGACGAACCGAAAC

Dsec ACCGCCTGCGCAATATGAGTCGGCTCATCTGCGCATATTCGACGGCGGACGAACCGAAAC

Dsim ACCGCCTGCGCAATATGAGTCGGCTCACCTGCGCATATTCGACGGCGGACGAACCGAAAC

Dmau CATACGCTCTTGCTCCAACGAATCCCTGGCCTTTTCCCGCGCTATGCAGGACCCAAATGC

Dsec CATACGCTCTTGCTCCAACGAATCCGTGGCCTTTTCCCGCGCTATGCAGGACCCAAATGC

Dsim CATACGCTCTTGCTCCAACGAATCCCTGGCCTTTTCCCGCGCTATGCAGGACCCAAATGC

Dmau TACCGATCAGGAACGCGCCGCTAAGCTTCGTGAGTGCAGTAG-GTCTCATCGAACA----

Dsec TACCGATCAGGAACGCGCCACTAAGCTTCGTGAG-GCAGTAGTGTCTCATCAGACATATG

Dsim TACCGACCAGGAACGCGCCGCTAAGCTTCGTGAA-GCAGTAGTGTCCCATCAGACATATG

**CG7362**

Dsim ATGCTACCTACGGGAAACAGATTTCCAAAGAATAATGGGAAAATAATGCCTCTAATTATC

DsimL ------------------------------------------------------------

Dmau ------------------------------------------------------------

Dsec ATGCTACCTACGGGAAACAGATTTCCAAAGAATAATGGGAAAATAATGCCTCTAATTATC

Dsim ATTAATAAATCGAGAGACAAGTCAACAAACCCGGCCGTCGAGTCAACAACTTGGTTGCGC

DsimL ------AAATCGAGAGACAAGTCAACAAACCCGGCCGTCGAGTCAACAACTTGGTTGCGC

Dmau ------AAATCGAGAGACAAGTCAACAAACCCGGCCGTCGAGTCAACAACTTGGTNGCGC

Dsec ATTAATAAATCGAGAGACAAGTCAACAAACCCGGCCGTCGAGTCAACAACTTCGTTGCGC

Dsim TTCAAGGCAGCCAAAGGAAAACGTTTCCTTTTGGCCAATGGATTGGATGGCGGCCAAACA

DsimL TTCAAGCAGCCCAAAGGAAAACGTTTCCTTTTGGCCAATGCATTAAATGGCGGCCAAACA

Dmau TTCAANGCAGCCAAAGGAAAACGTTTCCTTTNGGCCAATGGATNGGATGACNGCCAAACA

Dsec TTCAAGGCAGCCAAAGGAAAACGTTTCCTTTTGGCCAATGGATTGGATAGCGGGAAAACA

Dsim ATGCGTCCAGTTTGGACAAACATTTGCGGCAGCAAACCCATTTCATGCCCATTCCAACTT

DsimL ATGCGTCCAGTTTGGACAAACATTTGCGGCAGCAAACCCATTTCATGCCCATTCCAACTT

Dmau ATGCGTNCAGTTTGGACAAACATTTGCGGCAGCAAACCCATTTCATGCCCATTCCAACTT

Dsec ATGCGTCCAGTTTGGACAAACATTTGCGGCAGCAAACCCATTTCATGCCCATTCCAACTT

Dsim CCCACGTTGCACGGCAGCCTCAAAATTCAGATTTTTTTTAAATTTATCATATATTTGGTC

DsimL CCCACGTTGCACGGCAGCCTCAAAATTCAGATATTTATTAAATTTATCATATATTTGGTC

Dmau CCCACGTTGCACGGCAGCCTCAAAATTCAGATTTTTATTAAATTTATCATATATTTGGTC

Dsec CCCACGTTGCACGGCAGCCTCAAAATTCAGATTTTTATTAAATTTATCATATATTTGGTC

Dsim CGCATTACGCAAAATTTTTTTCAAAATTCGAAAATACGCCGGAAATCTTTGTCAGAGGGC

DsimL TGCATTACGCAAAATTTTTT-CAAAATTCGAAAATACGCCGGAAATCTTTGTCAGAGGGC

Dmau TGCATTACGCAAAATTTTTT-CAAAATTCGAAAATACGCCGGAAATCTTTGTCAGAGGGC

Dsec CGCATTACGCAAAATTTTTTTCAAAATTCGAAAATACGCCGGAAATCTTTGTCAGAGGGC

^

Dsim AAGCCTCTGCGTTACGTCACCATGTTCCAATTTTCCATTGAGCCT**TAG**ATTTCGCTGCTA

DsimL AAGCCTCTGCGTTGCGTCACCATGTCCCAATTTTCCATCAAGTTTTAGATTTCGCTGCTA

Dmau AAGCCTCAGCGTTGCGTCACCATGTCCCAATTTTCCATCAAGCCTTAAATTTCGCTGCTA

Dsec AAGCCTCTGCGTTGCGTCACCAT------------------GCCT**TAG**ATGTCGCTGCTA

Dsim TGCCGACAAATGCAAACGGAAAGTGCAACCCAAGAAGTTCCGGCTTGACCGTGGACCCTA

DsimL TGCTGACAAATGCAAACGGAAAGTGCAACCCAAGAAGTTCCGGCTTGACCGTGGACCCTA

Dmau TGCCGACAAATGCAAACGGAAAGTGCAACCCAAGAAGTTCCGGCTAGACCGTGGACCCTA

Dsec TGCCGACAAATGCAAACGGAAAGTGCAACCGAAGAAGTTCCGGCTAGACCGTGGACCCTA

Dsim CCTTTCCCAGCTGGACTACCAATCTCGTCTGCAGTTCCAAGCACCAGCTCTGAGGTTACC

DsimL CCTTTCCCAGCTGGACTACCAATCTCGTCTGCAGTTCCAAGCACCAGCTCTGAGGTTACC

Dmau CCTTTCCCAGCTGGACTACCAATCTCGTCTGCAGTTCCAAGCACCAGCTCTGAGGTTACC

Dsec CCTTTCCCAACTGGACTACCAATCTCGTCTGCAGTTCCAAGCACCAGCTCTGAGGTTACC

Dsim CCTCACCAGCATTATATGCACCATTGGACCCTCATCCAGCCAGCCTGAAGTTCTCCTTAA

DsimL CCTCACCAGCATTATATGCACCGTTGGACCCTCATCCAGCCAGCCCGAAGTTCTCCTTAA

Dmau CCTCACCAGCATTATATGCACCATTGGACCCTCATCCAGCCAGCCCAAAGTTCTCCTCAA

Dsec CCTCACCAGCATTATATGCACCGTTGGACCCTCATCCAGCCAGCCCGAAGTTCTCCTTAA

Dsim TCTCATTCATGCTGGGATGAAGGTGGTCCGATTGGACTTCTCCCACGGCACCCACGATTG

DsimL TCTCATTCATGCTGGGATGAAGGTGGTCCGATTGGACTTCTCCCACGGAACCCACGAATG

Dmau TCTCATTCATGCTGGGATGAAGGTGGTCCGATGGGACTTCTCCCACGGCACCCACGAATG

Dsec TCTCATTCATGCTGGGATGAAGGTGGTCCGATTGGACTTCTCCCACGGCACCCACGAATG

Dsim CCATTGCCAGGCAATACAGGCGGCACGTAAAGCCATCGCCATGTATGTGGAGGAAACGGG

DsimL CCATTGCCAGGCAATCCAGGCGGCACGTAAAGCCATCGCCATGTATGTGGAGGAAACGGG

Dmau CCATTGCCAGGCAATCCAGGCGGCACGTAAAGCCATCGCCATGTATGTGGAGGANACGGG

Dsec CCATTGCCAGGCAATCCAGGCGGCACGTAAAGCCATCGCCATGTATGTGGAGGAAACGGG

Dsim TCTTTCCAGGAGCTTGGCCATTGCACTGGACACCAAGGGTCCGGCAATCAATCCACAGGG

DsimL TCTTCTCAGATGCTTGGCCATTGCACTGGACACCAAGGGTCCGGCAATCAATCCACCGGG

Dmau TCTACCCAGAAGCTTGGCCATTGCCCTGGACACCAAGGGTGCGGCAGTCAATCCACAGGG

Dsec TCTTCCCAGATGCTTGGCCATTGCCCTGGACACCAAGGGTCCAGAAATCAATCCACAGGG

Dsim TGTAGCTGTTGATTTAAACGCCATAACCGAGCAAGACAAACTGGATCTCAAGTTTGGGGC

DsimL TGTAGCTATTGATTTAAACACCATAACCGAGCAAGATAAACTGGATCTCAAGTTTGGGGC

Dmau TGCAGCTGTTGATTTCAACGCCATAACCGAGCAAGACAAACTGGATCTCAAGTGTGGGGC

Dsec TGTACCTGTTGATTTAAACGCCATAACCGAGCAAGACAAACTGGATCTCAAGTTTGGGGC

Dsim GGATCAGAAGGTGGACATGGTATTCGCGTCGTTCATCCGCGATGCCAAAGCTTTGCAAGA

DsimL GGATCAGAAGGTGGACATGATATTCGCGTCGTTCATCCGCGATGCCAAAGCTTTGAAAGA

Dmau GGATCAGAAGGTGGACATGATCTTCGCGTCGTTCATCCGCGATGCCAAAGCTTNGCAAGA

Dsec GGATCAGAAGGTGGACATGATCTTCGCGTCGTTCATCCGCGATGCTAAAGCTTTGCAAGA

Dsim AATTCGCCAGGCACTGGGTCCATCAAGTGAGCACATAAAGATCATTTCCAAGATCGAAAG

DsimL AATTCGCCAGGCACTGGGTCCATCAAGTGAGCACATAAAGATCATTTCCAAGATCGAAAG

Dmau AATTCGCCAGGCACTGGGTCCATCAAGTGAGCACATAAAGATCATTTCCAAGATCGAAAG

Dsec AATTCGCCAGGCACTGGGTCCATCAAGTGAGCACATAAAGATCATTTCCAAGATAGAAAG

Dsim TCAACAGGCTCTGGCGAACATAGATGAGATAATCCGCGAATCCGATGGCATAATGGTGGC

DsimL TCAACAGGCTCTGGCGAACATAGATGAGATAATCCGCGAATCCGATGGCATAATGGTGGC

Dmau TCAACAGGCTCTGGCGAACATAGATGAGATAATCCGCGAATCCGATGGCATAATGGTGGC

Dsec TCAACAGGCTCTGGCGAACATAGATGAGATAATCCGCGAATCCGATGGCATAATGGTGGC

Dsim CCTTGGGAATATGGGCGACGAAATAGCACTGGAGGCTGTACCGCTGGCCCAGAAATCGAT

DsimL CCTTGGGAATATGGGCAACGAAATAGCACTGGAGGCTGTACCGCTGGCCCAGAAATCGAT

Dmau CCTTGGGAATATGGGCAACGAAATAGCACTGGAGGCTGTACCGCTGGCCCAGAAATCGAT

Dsec CCTTGGGAATATGGGCAACGAAATAGCACTGGAGGCTGTACCGCTGGCCCAGAAATCGAT

Dsim CGTGGCCAAGTGCAACAAAGTTGGAAAGCCTGTGATCTGTGCCAATCAAATGATGAATTC

DsimL CGTGGCCAAGTGCAACAAAGTTGGAAAGCCTGTGATCTGTGCCAATCAAATGATGAATTC

Dmau CGTGGCCAAGTGCAACAAAGTTGGAAAGCCTGTGATCTGTGCCAATCAAATGATGAATTC

Dsec CGTGGCCAAGTGTAACAAAGTTGGAAGGCCTGTGATCTGTGCCAATCAAATGATGAATTC

Dsim GATGATAACCAAGCCACGTCCCACTCGCGCCGAATCCTCTGATGTGGCAAACGCAATCTT

DsimL TATGATAACCAAGCCACGTCCCACACGCGCCGAATCTTCTGATGTGGCAAACGCAATCTT

Dmau GATGATAACCAAGCCACGTCCCACACGCGCGGAATCTTCTGATGTGGCAAACGCAATCTT

Dsec GATGATAACCAAGCCACGTCCCACACGCGCCGAATCTTCTGATGTGGCAAACGCAATCTT

Dsim GGATGGTTGTGATGCCCTTGTGTTGTCTGATGAAACGGCCAAGGGTAAGTACCCGGTGCA

DsimL GGATGGTTGTGATGCCCTTGTGTTGTCAGATGAAACGGCCAAGGGTAAGTACCCGGTGCA

Dmau GGATGGTTGTGATGCCCTTGTGTTGTCAGGTGAAACGGCCAAGGGTAAGTACCCGGTGCA

Dsec GGATGGTTGTGATGCCCTTGTGTTGTCTGATGAAACGGCCAAGGGTAAGTACCCGGTGCA

Dsim ATGTGTGCAGTGCATGGCCAGAATCTGCGCCAAGGTGGAGTCGGTTTTATGGTACGAGAG

DsimL ATGTGTGCAGTGCATGGCCAGAATCTGCGCCAAAGTCGAGTCCGTTTTATGGT-------

Dmau ATGTGTGCAGTGCATGGCCAGAATCTGCGCCAAGGTGGAGTCGGTTTTATGGTACGAGNG

Dsec ATGTGTGCAGTGCATGGCCAGAATCTGCGCCAAGGTGGAGTCGGTTTTATGGTACGAGAG
